# Identification of Three Classes of Heteroaromatic Compounds with Activity against Intracellular *Trypanosoma cruzi* by Chemical Library Screening

**DOI:** 10.1371/journal.pntd.0000384

**Published:** 2009-02-24

**Authors:** Esther Bettiol, Marie Samanovic, Andrew S. Murkin, Jayne Raper, Frederick Buckner, Ana Rodriguez

**Affiliations:** 1 Department of Medical Parasitology, New York University School of Medicine, New York, New York, United States of America; 2 Department of Biochemistry, Albert Einstein College of Medicine, Bronx, New York, United States of America; 3 Department of Medicine, University of Washington, Seattle, Washington, United States of America; University of California San Francisco, United States of America

## Abstract

The development of new drugs against Chagas disease is a priority since the currently available medicines have toxic effects, partial efficacy and are targeted against the acute phase of disease. At present, there is no drug to treat the chronic stage. In this study, we have optimized a whole cell-based assay for high throughput screening of compounds that inhibit infection of mammalian cells by *Trypanosoma cruzi* trypomastigotes. A 2000-compound chemical library was screened using a recombinant *T. cruzi* (Tulahuen strain) expressing β-galactosidase. Three hits were selected for their high activity against *T. cruzi* and low toxicity to host cells *in vitro*: PCH1, NT1 and CX1 (IC_50_: 54, 190 and 23 nM, respectively). Each of these three compounds presents a different mechanism of action on intracellular proliferation of *T. cruzi* amastigotes. CX1 shows strong trypanocidal activity, an essential characteristic for the development of drugs against the chronic stage of Chagas disease where parasites are found intracellular in a quiescent stage. NT1 has a trypanostatic effect, while PCH1 affects parasite division. The three compounds also show high activity against intracellular *T. cruzi* from the Y strain and against the related kinetoplastid species *Leishmania major* and *L. amazonensis*. Characterization of the anti–*T. cruzi* activity of molecules chemically related to the three library hits allowed the selection of two compounds with IC_50_ values of 2 nM (PCH6 and CX2). These values are approximately 100 times lower than those of the medicines used in patients against *T. cruzi*. These results provide new candidate molecules for the development of treatments against Chagas disease and leishmaniasis.

## Author Summary

Chagas disease is caused by infection with the protozoan parasite *Trypanosoma cruzi* and affects 16 million people in South and Central America. The disease starts with an acute phase where the parasite replicates rapidly and, if it remains untreated, is followed by a chronic phase, which can induce severe pathologies including cardiac insufficiency and megacolon, leading to death. Only two drugs with high toxicity exist to treat the acute phase of the disease and no drug is available for treatment of the chronic stage. We have screened a chemical library containing 2000 compounds to find molecules that inhibit the infection of host cells by *T. cruzi in vitro*. We found three different families of compounds that inhibit the parasite infection very efficiently, with low toxicity to host cells *in vitro*. We found that two of the compounds inhibit replication of the parasites, but the third one induces complete disintegration of the parasites inside host cells. This is especially interesting for the development of new drugs against the chronic stage of the disease, where parasites are intracellular and do not replicate actively.

## Introduction

Chagas disease or American trypanosomiasis is a devastating disease caused by the trypanosomatid protozoan *Trypanosoma cruzi*. It is endemic in 18 countries of Central and South America, putting 120 million of people at risk, with an estimated 16–18 million people currently infected [1]. The disease first manifests itself with an acute phase involving symptoms of swelling near the infection site, fever, fatigue, and enlarged lymphatic organs. It can then remain asymptomatic or manifest itself in a chronic form leading to cardiac insufficiency and megacolon. The two available drugs used to fight *T. cruzi* parasites during the acute stage are benznidazole (BZN) (Rochagan, Hoffmann-LaRoche) and nifurtimox (Lampit, Bayer). These drugs have toxic side effects and are not always effective. There is no drug available to treat the chronic stage of Chagas disease. Though some studies suggest that treatment with either BZN or nifurtimox decreases parasite load and slows disease progression, treatment of the chronic stage with these compounds is not officially recommended [2].

*T. cruzi* cases predominate in South America, but as migrant numbers increase in the USA, Canada and Europe, Chagas disease becomes a more widely spread public health problem, especially because BZN and nifurtimox are not approved by the countries' respective regulatory agencies and disease can be transmitted by contaminated blood donations. A need for development of new anti-*T. cruzi* compounds targeting the acute and/or chronic stages of the disease is therefore urgent.

The *T. cruzi* life cycle requires both an insect and a mammalian host. In the latter, the parasite development involves two stages: the amastigote form (intracellular parasites actively dividing within the cytoplasm of infected cells) and the trypomastigote form (free motile parasites that are released upon cell rupture into the blood and are able to infect cells) [3]. Compounds with curative properties will be efficient if they target either free trypomastigotes to inhibit the re-invasion of new cells, or intracellularly dividing amastigotes to prevent the release of new infective parasites.

*Leishmania* is a kinetoplastid parasite releated to *T. cruzi* and the causative agent of leishmaniasis, a disease whose manifestations in humans range from mild cutaneous and mucocutaneous lesions to fatal visceral infections. Among the many species responsible for cutaneous leishmaniasis, *L. major* of the Old World, is prevalent in Europe, Asia and Africa and *L. amazonensis* of the New World, extends from Southern Texas in North America to Brazil in South America. These two species diverged from each other 40–80 million years ago, leading to significant differences in host-parasite interactions and hence response to drugs [4]. Human infection initiates with the bite of a sandfly that deposits non-dividing metacyclic promastigotes into the host skin. The parasites are then taken up by professional phagocytes, differentiate to obligate intracellular amastigotes and multiply within an acidified phagolysosome, known as the parasitophorous vacuole. They eventually rupture the cell and spread further to uninfected cells. Therefore effective drugs should target the intravacuolar dividing parasites. Pentavalent antimony is still widely used to treat leishmaniasis, but drug resistance has appeared. Currently, the efficacy of liposomal Amphotericin B injected in mono- and combination therapies is being evaluated [5] and has displayed 90% of cure rates in combination with oral Miltefosine for visceral disease [6]. However, some cutaneous leishmaniasis are refractory and other drug treatments have 50% cure rates.

Screening libraries of chemical compounds against a standardized highly reproducible simple assay, or high throughput screening (HTS), offers an important tool in accelerating the discovery of new leads against parasitic diseases. This strategy's rationale is based on the assumption that screening of molecules with drug-like properties and highly diverse three-dimensional structures could allow the discovery of attractive new targets.

A transgenic *T. cruzi* strain expressing the reporter enzyme β-galactosidase (β-gal), also named LacZ, from *Escherichia coli* has been engineered by Buckner *et al.* [7]. This strain allows simple detection of parasite growth by measuring the β-gal activity, which correlates with parasite numbers. Other parasites expressing β-gal, such as *Toxoplasma gondii*, have been effectively used for screening compounds [8],[9]. The *T. cruzi* β-gal strain induces severe pathology *in vivo* [10], and it has been shown to grow *in vitro* similarly to control strains [7]. Beta-Gal *T. cruzi* were successfully used to screen compounds for activity against *T. cruzi* epimastigotes, which is the form found in the intestine of the insect host [11]. Compounds active against *Leishmania mexicana* and *Trypanosoma brucei* were also tested both on intracellularly replicating *T. cruzi* β-gal parasites and on contaminated blood [7].

In this study, we have optimized a whole-cell-based assay for HTS using the *T. cruzi* β-gal strain and screened a 2000-compound library to discover new molecules with activity against *T. cruzi*. We identified three compounds which inhibit intracellular replication of amastigotes in the nanomolar range and low toxicity on mammalian cells.

## Methods

### Parasite and mammalian cells

LLC-MK2 and NIH/3T3 cells were cultivated in DMEM supplemented with 10% FBS, 100 U/ml penicillin, 0.1 mg/ml streptomycin, and 0.292 mg/ml glutamine (Pen-Strep-Glut).

*T. cruzi* parasites from the Tulahuen strain stably expressing the β-gal gene (clone C4) [7] and from the Y strain were maintained in culture by infection of LLC-MK2 or NIH/3T3 cells every 5 or 6 days in DMEM with 2% FBS and 1% Pen-Strep-Glut. Bone marrow-derived macrophages were prepared from femurs of BALB/c mice (Taconic) and cultured for 7 days in DMEM supplemented with 10% FBS, Pen-Strep-Glut and 30% (v/v) L cell-conditioned medium as a source of CSF-1.

Trypomastigotes were obtained from the supernatant of infected cultures harvested between days 5 and 7. To remove amastigotes, trypomastigotes were allowed to swim out of the pellet of samples that had been centrifuged for 7 min at 2500 rpm. *L. major* strain Friedlin V1 (MHOM/JL/80/Friedlin) promastigotes were grown in medium M199 as previously described [12], and infective-stage metacyclic promastigotes were isolated from stationary 5-day old cultures by density centrifugation on a Ficoll gradient [13]. *L. amazonensis* IFLA/BR/67/PH8 strain promastigotes were maintained *in vitro* as previously described [14]. All cells and parasites were cultivated at 37°C in an incubator containing 5% CO_2_ and 95% air humidity, unless specified otherwise.

### *T. cruzi* growth inhibition assay

NIH/3T3 cells and parasites were harvested, washed once and resuspended in DMEM supplemented with 2% FBS and Pen-Strep-Glut. DMEM did not contain phenol red to avoid interference with the assay absorbance readings at 590 nM. Different numbers of NIH/3T3 cells were seeded in 96-well plates. After 3 h, compounds were added at the indicated concentrations and mixed by pipetting. BZN tablets (Rochagan, Roche) dissolved in DMSO and 4 µM Amphotericin B solution (Sigma-Aldrich) were used as positive controls. Different numbers of *T. cruzi* parasites were added in a final volume of 200 µl/well. After 4 days, 50 µl of PBS containing 0.5% of the detergent NP40 and 100 µM Chlorophenol Red-β-D-galactoside (CPRG) (Fluka) were added. Plates were incubated at 37°C for 4 h and absorbance was read at 590 nm using a Tecan Spectra Mini plate reader.

To calculate the Z′ factor, we used the formula described by Zhang *et al.* [15]: Z′ = 1−[(3σ_c+_+3σ_c−_) / |μ_c+_−μ_c−_|] where σ_c+_ = standard deviation (SD) of positive control, σ_c−_ = SD of negative control, μ_c+_ = mean of positive control, μ_c−_ = mean of negative control. Subsequently, the best ratio was used for all growth inhibition assays (50.000 cells and parasites, multiplicity of infection (MOI) 1∶1).

To determine IC_50_ values, β-gal activity (Abs_590_) was plotted against compound concentration for each compound. The IC_50_ was determined as the concentration at which the activity (absorbance) was half that in the absence of compound. Mean IC_50_ values are the average of independent experiments performed in triplicate on three different days.

### Chemical library and screen protocol

Two thousand compounds in dimethyl sulfoxide (DMSO) from the DIVERSet library (ChemBridge Corporation, San Diego, CA) were screened at 25 µg/ml in 96-well plates (80 compounds per plate). Each plate also contained triplicate wells of negative control (no compounds), positive control (4 µM Amphotericin B) and 1% DMSO (vehicle). Selected hits among the screened compounds include *N*′-{[5-(2,3-dichlorophenyl)-2-furyl]methylene}-2-pyridinecarbohydrazide (hydrazide 1; **PCH1**), 2-(3-nitro-1*H*-1,2,4-triazol-1-yl)-*N*-{3-nitro-5-[3-(trifluoromethyl)phenoxy]phenyl}acetamide (nitrotriazole 1; **NT1**) and 1-[6-(4-chloro-3,5-dimethylphenoxy)hexyl]-1*H*-imidazole (chloroxylenol 1; **CX1**).

Chemically related compounds were also ordered from ChemBridge Corporation and include *N*′-{[5-(2,3-dichlorophenyl)-2-furyl]methylene}nicotinohydrazide (**PCH2**), *N*′-{[5-(2,3-dichlorophenyl)-2-furyl]methylene}isonicotinohydrazide (**PCH3**), 4-bromo-*N*′-{[5-(2,3-dichlorophenyl)-2-furyl]methylene}benzohydrazide (**PCH4**), *N*′-{[5-(3-chlorophenyl)-2-furyl]methylene}-2-pyridinecarbohydrazide (**PCH5**), *N*′-{[5-(2-chlorophenyl)-2-furyl]methylene}-2-pyridinecarbohydrazide (**PCH6**), *N*′-{[5-(3,4-dichlorophenyl)-2-furyl]methylene}-2-pyridinecarbohydrazide (**PCH7**), *N*′-{[5-(3-chloro-4-methoxyphenyl)-2-furyl]methylene}-2-pyridinecarbohydrazide (**PCH8**), *N*′-{[5-(2,5-dichlorophenyl)-2-furyl]methylene}benzohydrazide (**PCH9**), *N*′-{[5-(2-chlorophenyl)-2-furyl]methylene}nicotinohydrazide (**PCH10**), *N*-(3-methoxy-5-nitrophenyl)-2-(3-nitro-1*H*-1,2,4-triazol-1-yl)acetamide (**NT2**), *N*-[3-nitro-5-(3-pyridinyloxy)phenyl]-2-(3-nitro-1*H*-1,2,4-triazol-1-yl)acetamide (**NT3**), *N*-{3-[(5-chloro-3-pyridinyl)oxy]-5-nitrophenyl}-2-(3-nitro-1*H*-1,2,4-triazol-1-yl)acetamide (**NT4**), 2-(3-nitro-1*H*-1,2,4-triazol-1-yl)-*N*-[3-(trifluoromethyl)phenyl]acetamide (**NT5**), *N*-[2-chloro-5-(trifluoromethyl)phenyl]-2-(3-nitro-1*H*-1,2,4-triazol-1-yl)acetamide (**NT6**), *N*-[4-chloro-2-(trifluoromethyl)phenyl]-2-(3-nitro-1*H*-1,2,4-triazol-1-yl)acetamide (**NT7**), *N*-[2-chloro-5- (trifluoromethyl)phenyl]-4-(3-nitro-1*H*-1,2,4-triazol-1-yl)butanamide (**NT8**), 4-(3-nitro-1*H*-1,2,4-triazol-1-yl)-*N*-[2-(trifluoromethyl)phenyl]butanamide (**NT9**), 1-[5-(4-chloro-3,5-dimethylphenoxy)pentyl]-1*H*-imidazole (**CX2**), 1-[4-(4-chloro-3,5-dimethylphenoxy)butyl]-1*H*-imidazole (**CX3**), 1-[6-(4-chloro-2,6-dimethylphenoxy)hexyl]-1*H*-imidazole (**CX4**), 1-[5-(4-chloro-2,6-dimethylphenoxy)pentyl]-1*H*-imidazole (**CX5**) and 1-[4-(4-chloro-2,6-dimethylphenoxy)butyl]-1*H*-imidazole (**CX6**). The derivatives **PCH2**–**PCH10** were chosen with >80% similarity to **PCH1**, **NT2**–**NT9** with >85% similarity to **NT1** and **CX2**–**CX6** with >90% similarity to **CX1**.

### *T. cruzi* lysis assay

Trypomastigotes were rinsed once and plated in 96-well plates at 100,000/well with the compounds in a final volume of 200 µl of DMEM without phenol red supplemented with 2% FBS, Pen-Strep-Glut and 100 µM CPRG. Plates were incubated for 24 h at 37°C and absorbance was read at 590 nm.

### Cytotoxicity assay

Cells (NIH/3T3 or HepG2) were washed and plated at a density of 50,000 cells/well of 96-well plates in 200 µl and allowed to adhere for 3 h. Twenty-four hour assays were done in DMEM without phenol red supplemented with 10% FBS and Pen-Strep-Glut, while 4-day assays were done in the same medium containing 2% FBS. Drugs were added and mixed. After 1 or 4 days, 20 µl of Alamar Blue (Biosource, Invitrogen) was added. Plates were incubated for 4 h (HepG2) or 6 h (NIH/3T3) at 37°C and fluorescence was read using a Labsystems Fluoroskan II plate reader (excitation: 544 nm, emission: 590 nm) .

To determine TC_50_ values, fluorescence was plotted against inhibitor concentration. TC_50_ was determined as the concentration at which cytotoxicity (fluorescence) was half that in the absence of inhibitor.

### Invasion and development assays

Fifty thousand NIH/3T3 cells were seeded on sterile glass coverslips in 12-well plates and allowed to adhere overnight. Five million parasites were added (MOI 100∶1) and allowed to infect for 2 h in DMEM+2% FBS and Pen-Strep-Glut. Parasites were rinsed out three times with PBS. Infected cells were further incubated and fixed for 15 min with 4% paraformaldehyde at the times indicated.

### Immunofluorescence assay

Fixed cells on coverslips were rinsed with PBS, permeabilized for 15 min in PBS with 0.1% Triton X-100 (Sigma-Aldrich). After blocking for 20 min in PBS with 10% goat serum, 1% bovine serum albumin, 100 mM glycine and 0.05% sodium azide, cells were incubated for 1 h at room temperature with a polyclonal rabbit anti-*T. cruzi* (gift from Dr B. Burleigh, Harvard School of Public Health, Boston, MA) at 1∶2000 dilution. After rinsing, an Alexa Fluor® 488 goat anti-rabbit IgG secondary antibody (Molecular Probes, Invitrogen) was added for 1 h at a 1∶800 dilution. DNA was stained with DAPI and coverslips were mounted on Mowiol. To determine the number of parasites per infected cell, between 200 and 300 infected cells per coverslip were scored in triplicate samples using an inverted Olympus IX70 microscope with a 60× oil objective. Data are presented as mean±standard deviation. Images were taken with the same microscope.

### *Leishmania* growth inhibition assay

Adherent bone marrow-derived macrophages were harvested in cold DMEM+0.5 mM EDTA and seeded into an 8-well Lab-Tek II chambered coverglass (Nalge Nunc International, Naperville, IL) at a concentration of 50,000 cells/chamber 24 h before being used for infections.

*L. major* and *L. amazonensis* parasites were opsonized for 30 min by incubation in DMEM containing 4% BALB/c serum and then allowed to invade macrophages in 200 µl DMEM supplemented with 10% FBS and Pen-Strep-Glut, at a MOI of 3 parasites per macrophage for 2 h at 33°C (5% CO_2_, 95% air humidity) [16],[17]. Thereafter, non-phagocytosed parasites were washed off, and the cultures were further incubated in 300 µl of medium in the presence or the absence of drugs at the indicated concentration for 3 days for *L. amazonensis* and 5 days for *L. major*. Medium was changed and drugs were added again at the same concentration on day 2 post-infection. Intracellular parasites were assessed after staining with DAPI (3 µM) by fluorescence microscopy. The total number of amastigotes/500 macrophages was counted in each well. Kruskal-Wallis test was used to analyze the data, followed by a Dunn's post-comparison test.

## Results

### Optimization of a 4-day assay for screening

Our first goal was to optimize a simple and reliable assay for HTS in 96-well format to quantify *T. cruzi* trypomastigotes' infection of host cells. This type of assay would allow for the identification of compounds that inhibit either free extracellular trypomastigotes or intracellularly dividing amastigotes. The primary protocol for β-gal-expressing *T. cruzi* trypomastigotes of the Tulahuen strain infecting NIH/3T3 cells [7] was modified to shorten the incubation time of the assay. This is an important parameter because short incubation times decrease medium evaporation and lessen concerns about compound stability.

The Z′ factor is a statistical parameter used to assess the reproducibility and quality of HTS assays by taking into account the signal dynamic range and the data variation [15]. Assays with Z′ factors between 0.5 and 1 are considered appropriate for HTS. To determine which parasite∶cell ratio was required to shorten the incubation time, different concentrations of host cells (NIH/3T3) and trypomastigotes were tested with or without the well-characterized anti-trypanosomal compound Amphotericin B. It was found that 50,000 host cells and 50,000 parasites per well incubated for 4 days yielded a high and reproducible signal. The mean Z′ factor of independent experiments performed in sextuplicate on three different days was 0.834 (±0.018).

### Screening of a 2000-compound library

**Figure 1:**
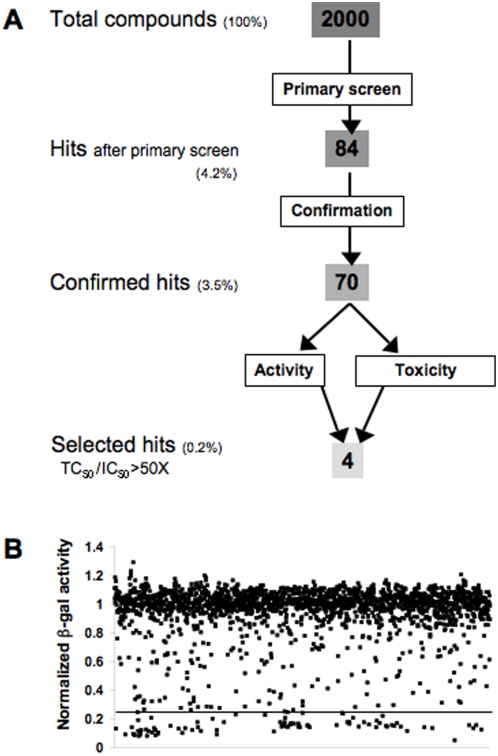
Screening steps statistics and distribution of compounds. A. Schematics of the screening steps with number of compounds and percentage of total compounds at each step. B. Distribution of relative β-gal activity values of the 2000 compounds (after normalization to the median of each assay plate). The line shows the hit selection cutoff (as the average of the normalized hit threshold per plate).

To discover new compounds with anti-*T. cruzi* activity, a library of 2000 compounds (DIVERSet from Chembridge Corporation) was screened, initially at 25 µg/ml in single wells. This library contains compounds from a larger library (EXPRESS-Pick Collection) that are chosen for maximum pharmacophore diversity based on 3D conformation and drug-like properties. We hypothesized that adding test compounds to cells at the same time than parasites would allow the detection of compounds with both anti-free trypomastigotes and anti-intracellular growth activities. Primary screen concentration was 25 µg/ml, which corresponds to a range of 42 to 112 µM, based on molecular weights from 223 to 587. The threshold for selecting hits was set as the average of positive controls (Amphotericin B 4 µM) plus two times the standard deviation. The screening steps are schematically illustrated in Fig. 1A. Eighty-four primary hits were obtained out of the 2000 compounds, as displayed in Fig. 1B, which represents the distribution of the normalized absorbance readings of the 2000 compounds. After retesting in exactly the same conditions, 70 hits were confirmed (3.5% of the total) (data not shown).

### Selection of hits

Our next goal was to select amongst the 70 confirmed hits the best candidates for further investigation. To this aim, compounds with high anti-trypanosomal efficacy and low toxicity to host cells were selected. The anti-trypanosomal activity of the 70 confirmed hits was first tested at six different concentrations from 25 µg/ml (51–110 µM depending on compound molecular weight) to 8 ng/ml (16–35 nM). In parallel, the toxicity of these compounds was tested in different concentrations with a 4-day assay on NIH/3T3 cells using Alamar Blue (data not shown). Fifty-nine of the 70 hits lost completely their activity at 5 µg/ml and were discarded.

**Figure 2:**
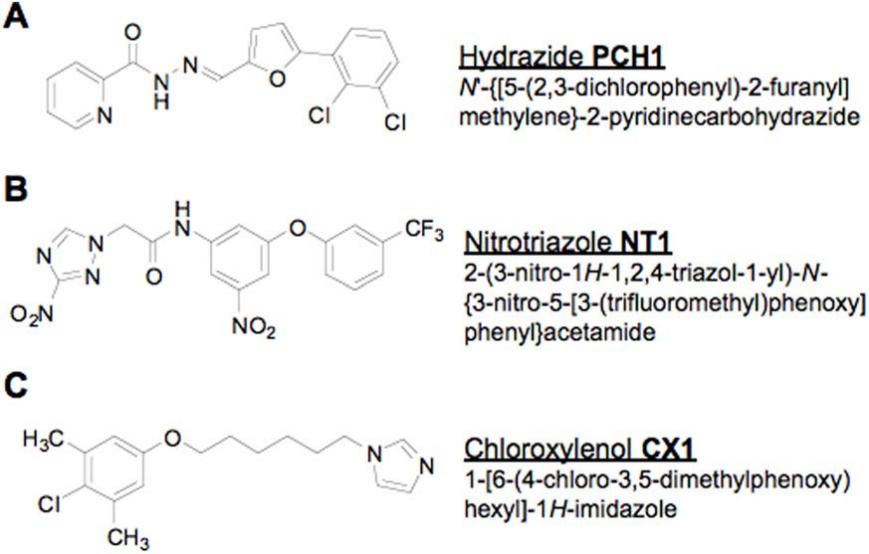
Three selected hits: chemical names, abbreviations and molecular structures.

Out of the 11 remaining hits, three compounds with the highest anti-trypanosomal activity and low toxicity levels were selected for further characterization: **PCH1**: *N*′-{[5-(2,3-dichlorophenyl)-2-furyl]methylene}-2-pyridinecarbohydrazide; **NT1**: 2-(3-nitro-1*H*-1,2,4-triazol-1-yl)-*N*-{3-nitro-5-[3-(trifluoromethyl)phenoxy]phenyl}acetamide; **CX1**: 1-[6-(4-chloro-3,5-dimethylphenoxy)hexyl]-1*H*-imidazole (Fig. 2). These three compounds have at least 50-fold higher toxicity levels (TC_50_) versus anti-trypanosomal activity (IC_50_). The eight other hits that retained activity at 5 µg/ml (described in Fig. S1) were not investigated further because of their low activity and/or high toxicity.

**Figure 3:**
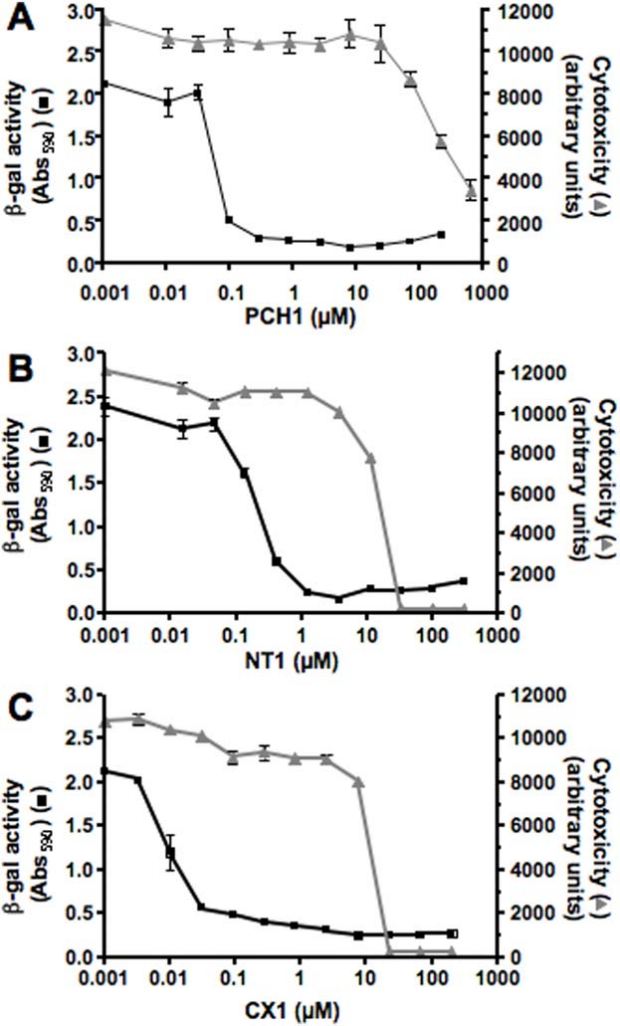
Dose-response curves of selected hits and cytotoxicity on NIH/3T3 cells. A. PCH1; B. NT1 and C. CX1. Each compound was tested for *T. cruzi* growth inhibition (black curves, left axis). 50.000 NIH/3T3 cells were incubated with 50.000 β-gal *T. cruzi* parasites per well in the presence of each compound and β-gal activity was assayed at 4 days. Cytotoxicity of each compound on NIH/3T3 cells was also assayed (grey curve, right axis), by incubating 50.000 cells in the presence of compounds. Viability was assayed by incubating cells on day 4 with Alamar Blue for 6 h and measuring fluorescence. Data are displayed as the mean±standard deviation of triplicate wells from one of three independent experiments.

Precise IC_50_ and TC_50_ values of the selected hits were calculated from dose-response curves (Fig. 3). The mean IC_50_ values (Table 1) of all three compounds are lower than 1 µM, with compounds **PCH1** and **CX1** having IC_50_ values in the low nanomolar range (54 and 23 nM, respectively). Under these assay conditions, the IC_50_ of BZN was found to be 1.15 µM±0.08 (data not shown), consistent with the value of 1.5 µM reported by Buckner *et al.* [7].

**Table 1:** IC_50_ and TC_50_ of selected hits.

	PCH1	NT1	CX1
IC_50_ Intracellular growth	54±10 nM	190±40 nM	23±7 nM
TC_50_ NIH/3T3 cells at 4 days	>300 µM	14±6 µM	16±6 µM
	*>5500×	*74×	*690×
TC_50_ HepG2 cells at 24 h	>300 µM	30±10 µM	57±8 µM
	*>5500×	*160×	*2400×
TC_50_ HepG2 cells at 4 days	66±7 µM	7.6±0.3 µM	12±4 µM
	*1200×	*40×	*530×
IC_50_ Free trypomastigotes	240±60 µM	6.8±0.9 µM	13.5±1.0 µM

*: Ratio of TC_50_ to IC_50_. Data are displayed as mean±SD of three independent experiments performed in triplicate.

To characterize the toxicity profiles of the three compounds further, cytotoxicity assays were performed with HepG2 cells, a human hepatoma cell line commonly used for *in vitro* testing of toxicity [18]. Cells were incubated with compounds for 24 h or 4 days. Mean TC_50_ values are displayed in Table 1. The ratio of TC_50_ to IC_50_ was again over 500 at both time points tested for **PCH1** and **CX1**. The TC_50_ of **NT1** was more than 150-fold greater than its IC_50_ at 1 day, but decreased to only 40-fold at 4 days.

### Mechanism of action of the selected compounds

Our next goal was to determine which stage of parasite development was inhibited by these compounds. To assess if the observed effect of compounds was due to direct lysis of free trypomastigotes before they even invaded cells, we performed a lysis assay in which 100,000 parasites were incubated for 24 h in the presence of increasing concentrations of the selected compounds and the β-gal substrate CPRG. In this assay, β-gal activity increases proportionally to the number of parasites that are lysed by the compound, releasing β-gal in the medium. The IC_50_ was in the micromolar range for all compounds as shown in Table 1, suggesting that the mechanism of the inhibition observed during infection of host cells was not due to a direct effect of the compounds on free trypomastigotes.

We next investigated which stage of host cell infection by *T. cruzi* trypomastigotes was inhibited by each of the compounds. To analyze the effect of the compounds in host cell invasion, we incubated NIH/3T3 cells for 2 h with trypomastigotes at the IC_100_ concentration. After thorough rinsing, fixation and staining of parasites, we did not find any significant difference with controls (data not shown).

**Figure 4:**
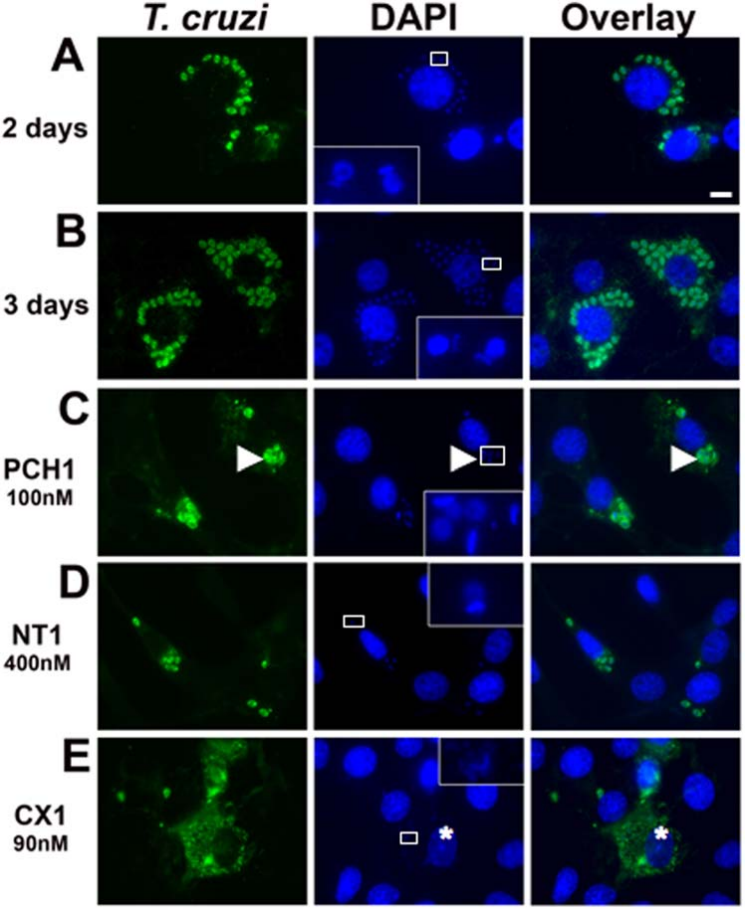
Compounds PCH1, NT1 and CX1 affect the morphology of intracellularly growing *T. cruzi*. Compounds were added after cells had been infected for 2 h and extracellular β-gal *T. cruzi* parasites had been removed by washing. Cells were fixed after 2 or 3 days, stained with an anti-*T. cruzi* antibody and DAPI to visualize DNA. Insets show a higher magnification of the parasite nucleus and kinetoplast. In control at 2 days (panel A) and at 3 days (panel B), multiple amastigotes homogenous in size and shape could be observed throughout the cytoplasm of infected cells. Upon treatment with 100 nM PCH1 (panel C), larger amastigotes containing multiple disorganized nuclei and kinetoplasts were observed (arrowheads). Treatment with NT1 at 400 nM (panel D) inhibited replication of parasites without altering their overall morphology. Cells treated with CX1 at 90 nM (panel E) displayed pycnotic nuclei (star) and debris of parasites protein and DNA throughout the cytoplasm. Scale bar: 5 microns.

Next, we assessed if compounds were interfering with intracellular proliferation of amastigotes within mammalian cells. We infected cells for 2 h, rinsed away the remaining free trypomastigotes and, after adding the compounds at the IC_100_ concentrations, we incubated cells for 2–3 days to allow for amastigote proliferation. In control cells, amastigotes homogenous in size were distributed throughout the cytoplasm of the host cells and kinetoplasts were observed closely apposed to the nucleus of parasites (Fig. 4A at 2 days and Fig. 4B at 3 days).

Upon treatment with **PCH1**, the morphology of parasites was severely affected (Fig. 4C). We observed larger amastigotes containing multiple nuclei and kinetoplasts, which were disorganized and had lost their normal 3-dimensional relationship. These results suggest that **PCH1** induces a defect in cell division.

Treatment with **NT1** resulted in infected cells containing only a few amastigotes of average size with apparently normal nucleus and kinetoplast (Fig. 4D), suggesting that this compound interferes with proliferation of amastigotes without affecting their morphology.

**CX1** induced parasite death, as observed by the decrease of structures clearly identifiable as amastigotes. Parasite proteins and DNA were observed all throughout the cytoplasm, suggesting that amastigotes were lysed. Moreover, the nucleus of the host cell containing parasites debris was often pyknotic, suggesting that death of the parasite was inducing death of the host cell (Fig. 4E).

**Figure 5:**
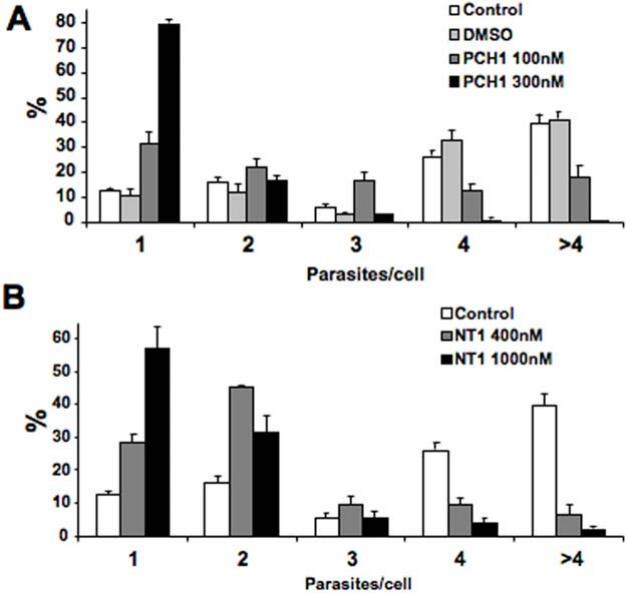
PCH1 and NT1 inhibit intracellular replication of β-gal *T. cruzi*. A. PCH1; B. NT1. Cells were incubated with *T. cruzi* trypomastigotes for 2 h, after which compounds were added at indicated concentrations and incubated for 2 days. Parasites were stained and the number of parasites per infected cell was counted. 200–300 cells/well were scored. Data represents the average values±standard deviation obtained from triplicate wells of a representative experiment out of three.

We also quantified the number of parasites per infected cell, confirming that both **PCH1** and **NT1** induced a growth arrest of intracellular *T. cruzi* (Fig. 5). In cultures treated with these compounds, the majority of infected cells contain only one or two parasites while in control cultures the majority of cells contain 4 or more parasites. The number of parasites per infected cell could no be quantified after treatment with **CX1** because no parasite structures were clearly visible. Of note, presence of multiple parasites within a cell can denote either amastigotes that have divided or a cell that has been infected by several trypomastigotes.

When the compounds were added 2 days after infection, similar phenotypes were observed: **PCH1** induced major defects in cell division at the IC_100_ and parasite lysis at higher doses. **NT1** had a trypanostatic effect. **CX1** induced parasite lysis and host cell apoptosis in concentrations as low as 90 nM (data not shown).

### Effect of PCH1, NT1 and CX1 on *T. cruzi* Y strain

**Figure 6:**
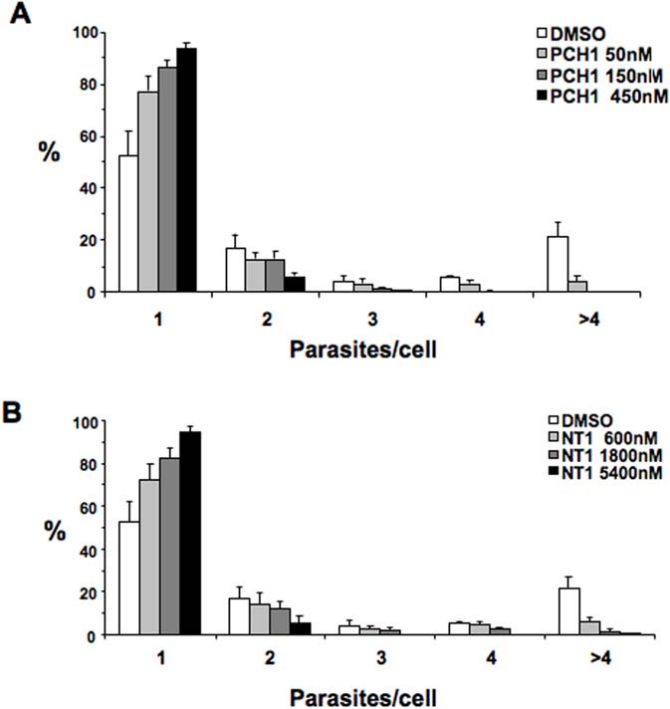
PCH1 and NT1 inhibit intracellular replication of *T. cruzi* Y strain. A. PCH1; B. NT1. Cells were incubated with Y strain trypomastigotes for 2 h, after which compounds were added and incubated for 2 days. Parasites were stained and the number of parasites per infected cell was counted. 200–300 cells/well were scored. Data represents the average values±standard deviation obtained from triplicate wells of a representative experiment out of two.

We also confirmed the effect of **PCH1**, **NT1** and **CX1** on the infection by *T. cruzi* trypomastigotes of the Y strain. We performed the same development assay and quantified the number of parasites per infected cell for **PCH1** (Fig. 6A) and **NT1** (Fig. 6B). As described above for the Tulahuen strain, **CX1** induced parasite lysis with morphological changes that prevented this type of quantification.

### Effect of compounds on growth of *L. major* and *L. amazonensis* in macrophages

**Figure 7:**
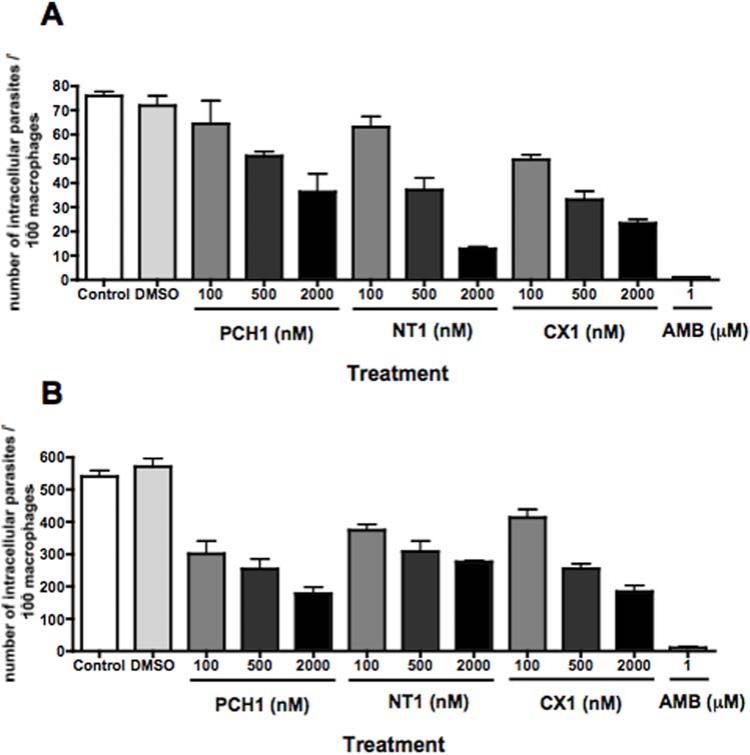
Intracellular *Leishmania* are sensitive to PCH1, NT1 and CX1. Bone marrow-derived macrophages were infected with either *L. major* (A) or *L. amazonensis* (B) parasites at a multiplicity of infection of 3∶1 for 2 h, before addition of the different compounds at the indicated concentrations. Infected macrophages were incubated for 5 (A) or 3 days (B), before the preparations were stained with DAPI and the number of parasite nuclei was counted. AMB, Amphotericin B. Data represents the average values obtained from triplicate wells of a representative experiment out of two.

To evaluate the effect of these three compounds on another intracellular kinetoplastid, we tested them against *L. major* and *L. amazonensis* parasites. In the vertebrate host, *Leishmania* parasites are intracellular and reside mostly within macrophages inside phagolysosomes. Therefore, we added a range of compound concentrations 2 h post-infection of macrophages with metacyclic promastigotes. A high dose of Amphotericin B (1 µM) was used as a positive control (IC_50_ = 0.1 µM, [19]). Five days post-infection with *L. major*, which resides in individual phagolysosomes, we observed a reduction in the number of intracellular parasites with the three compounds (Fig. 7A). We observed a reduction in parasite burden at the highest chemical concentration (2 µM) of about 50% for **PCH1**, 80% for **NCT1** (p<0.05), and 70% for **CX1** (p<0.05). To evaluate the effect of these compounds on intracellular *L. amazonensis*, which resides in large communal phagolysosomes, we repeated this experiment by adding a range of concentrations 2 h post-infection of macrophages for 3 days (Fig. 7B). The number of intracellular *L. amazonensis* parasites decreased in presence of each of the 3 compounds, reducing the parasite burden by 70% for **PCH1** (p<0.5), 50% for **NCT1** (p<0.5), and 70% for **CX1** (p<0.5) at 2 µM.

### Effect of chemically related compounds on *T. cruzi*

**Figure 8:**
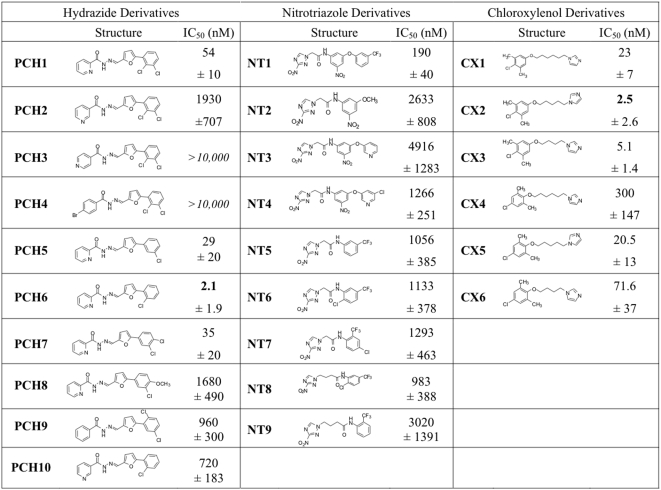
Inhibition of *T. cruzi* growth by PCH1, NT1, CX1 and their derivatives. Each compound was tested for *T. cruzi* growth inhibition to determine IC_50_ values. Data are displayed as mean±standard deviation of three independent experiments.

To assess if chemical modification of the compounds would improve their inhibitory effect on *T. cruzi* parasite growth and help us identify which parts of the molecules are important for their activity, we tested the activity of compounds chemically related to **PCH1**, **NT1** and **CX1** that were commercially available. These compounds were identified using the hit2lead website (https://www.hit2lead.com) and tested for activity against *T. cruzi* trypomastigote infection. The IC_50_ values for these compounds were determined and compared to their parental compounds (Fig. 8). We found that, while some of the chemical modifications caused a decrease of anti-trypanosomal activity, others resulted in increased efficacy. Interestingly, we found three compounds, **PCH6**, **CX2** and **CX3**, with significantly higher activity compared to their parental structures, with IC_50_ values of 2.1, 2.5 and 5.1 nM respectively (TC_50_ values of **PCH6** and **CX2** are 18.5 and 19.5 µM, respectively).

More specifically, for **PCH1**, the pyridine nitrogen was varied from *ortho* (**PCH1**) to *meta* (**PCH2**) and *para* (**PCH3**) positions, resulting in 35-fold and >200-fold increases in IC_50_, respectively. Substitution of the pyridine ring with a *para*-bromophenyl group as in **PCH4** also abolished activity, further reinforcing the importance of an *ortho*-nitrogen within the ring. Modifications to the chlorophenyl group explored the role of chloride substituents on this ring. Removal of the chloride at the *ortho* position as in **PCH5** did not alter the effect, whereas removal of the *meta*-chloride as in **PCH6** actually resulted in more potent inhibition (IC_50_ = 2.1 nM). Whereas repositioning the *ortho*-chloride of **PCH1** to the *para* position as in **PCH7** did not affect the effect, replacing this chloride with a methoxy group as in **PCH8** gave poorer inhibition (IC_50_ = 1.6 µM), indicating that electron-donating and/or steric properties are detrimental at this position. Combinations of modifications to the pyridine and chlorophenyl rings in **PCH9** and **PCH10** resulted in decreased efficacy; however, comparison of **PCH10** to the other *meta*-pyridine derivative **PCH2** again indicates that improved inhibition results from removal of the *meta*-chloride substituent, as was observed with **PCH1** and **PCH6**.

Chemical variations of **NT1** maintained the 2-nitrotriazole moiety of the parent while containing modifications in the linker, nitrophenyl and trifluorotoluyl groups (Fig. 8). These modifications include the removal of the latter aromatic ring as in **NT2** or replacement with a pyridine ring as in **NT3**, reintroduction of a substituent *meta* to the oxygen (**NT4**), removal of the nitrophenyl ring as in **NT5** or introduction of another electron-withdrawing substituent as in **NT6** and **NT7** or increasing the linker length between the nitrotriazole and carboxamide group and placing the trifluoromethyl group of **NT8** at the *ortho* ring position as in **NT9**. The IC_50_ values obtained for the modified compounds were all similar, suggesting that a variety of substituents are tolerated.

To explore the importance of the additional substituents and of the positioning of the two aromatic rings of **CX1**, analogues **CX2**–**CX6** were assayed for growth inhibition as well (Fig. 8). Truncation of the alkyl linker from six to five or four carbons in **CX2** and **CX3**, respectively, revealed a preference for the pentyl linker, yielding an IC_50_ of 2.5 nM. However, the butyl linker in **CX3** also gave IC_50_ lower than the parent compound **CX1** (5.16 nM versus 23 nM). When the positioning of the methyl groups on the chloroxylenol group of **CX1** was modified as in **CX4**, the effect was adversely affected, raising the IC_50_ to 300 nM. However, variation in the linker length as in **CX5** and **CX6** again revealed a similar pattern of preference, as the pentyl linked **CX5** also displayed the most potency among the 4-chloro-2,6-xylenol series, with an IC_50_ of 20.5 nM.

## Discussion

New compounds are desperately needed to fight efficiently *T. cruzi*, the parasite responsible for Chagas disease. To this aim, we optimized a simple and straight-forward assay that allows the HTS of compounds against *T. cruzi* replicating within mammalian cells. We tested 2000 compounds from the DIVERSet chemical library. This compound library has been useful to discover inhibitors of matrix metalloproteinase-9 in a whole cell assay [20] and to find inhibitors of the ribonucleic activity of angiogenin using a cell-free system [21]. However, to our knowledge this is the first time this library has been used to discover new anti-microbials.

After screening the 2000 compounds, 70 confirmed hits (3.5%) were obtained. The rate of hits was relatively high, probably due to two main reasons: (1) the high concentration of compounds used for primary screening (25 µg/ml) and (2) because any compounds that are toxic to mammalian cells would also be scored as hits in this experimental design, since they would affect the host cells that are required for parasite replication. After a secondary screening to eliminate these false positive hits and select the most effective compounds, three potential candidates (0.15% of all compounds) were identified that were active in the nanomolar range at the stage of intracellular replication of the *T. cruzi* parasites.

The three hits we selected had IC_50_ values in the low-nanomolar range and low toxicity on mammalian cells. Although HepG2 cells have a limited drug metabolism activity to assess toxicity of metabolites [22], they are a useful model as a primary toxicity screen due to their human origin and ease of use [18]. Interestingly, although the selected hits had IC_50_ values in the low-nanomolar range when tested on intracellular replication of parasites, they were only active on free trypomastigotes at higher concentrations that were similar to or above the TC_50_ on mammalian cells. Therefore, it appears that our screening assay favors the selection of drugs that are effective against intracellular replication of parasites but not active on free trypomastigotes. This is probably a consequence of adding compounds and trypomastigotes simultaneously to host cells, a procedure that would not allow enough time for compounds with activity against free trypomastigotes to prevent completely invasion of host cells.

One of the compounds that we have investigated, **NT1**, has an IC_50_ of 190 nM on the β-gal *T. cruzi* strain. **NT1** also displayed activity on the Y strain, but at higher concentrations. Interestingly, when tested against *L. major* and *L. amazonensis* **NT1** had a dose-dependant anti-leishmanial effect on the intracellular form of the parasites. **NTI** was potent against *L. major* and *L. amazonensis* with an estimated IC_50_ of ∼500 nM.

This compound inhibited *T. cruzi* amastigote replication within host cells, but we did not observe amastigote lysis at 2–3 days. Its effect might therefore be more trypanostatic than trypanocidal. The toxicity of **NT1** on mammalian cells was between 40- and 159-fold depending on the type of mammalian cells and the duration of the cytotoxicity assay. This is a relatively high toxicity and might therefore be an issue for further development of this compound.

**NT1** is chemically similar to BZN in that they both contain an acetamide group linked to a nitro-substituted, heteroaromatic five-membered ring (triazole and imidazole, respectively). **NT1** is also chemically related to the approved anti-fungal agent fluconazole, as it contains a triazole ring, of which fluconazole has two. Fluconazole has an IC_50_ against *T. cruzi in vitro* of 8 µM [7],[23], but its activity in mice models of *T. cruzi* infection has not been confirmed [24]. Moreover, fluconazole has been used with some success against cutaneous leishmaniasis caused by *L. major*, although some geographically distinct species such as *L. tropica* are refractory.

Upon testing of chemical analogues of **NT1** that preserve the nitrotriazole moiety but include a variety of aryl and aryl ether substituents, we found that these variations did not modify strongly the anti-trypanosomal effect. It is therefore likely that the pharmacophore is the nitrotriazole group acting through a non-targeted mechanism, like BZN.

Another compound identified in the initial screen, **CX1**, possesses imidazole and phenyl rings, similar to BZN but without a nitro substituent on the imidazole group and with chloride and methyl groups on the phenyl ring (i.e., 4-chloro-3,5-xylenol). It is not clear whether **CX1** and BZN share the same target in *T. cruzi*. Comparison of the anti-*T. cruzi* activity of **CX1** and BZN side by side revealed that the IC_50_ of **CX1** is 50 times lower than that of BZN (23 nM versus 1.15 µM). **CX1**'s dose effect on the Y strain of *T. cruzi* was similar to the β-gal-expressing Tulahuen strain, suggesting that the IC_50_ is close for the two strains. Additionally, intracellular *L. major* and *L. amazonensis* are sensitive **CX1**. Indeed, it significantly reduced the *L. major* and *L. amazonensis* parasite burden by 70% at a concentration of 2 µM, and had estimated IC_50_ of ∼500 nM against both pathogens.

Numerous studies have been performed trying to modify imidazole derivatives to decrease their toxicity profile, which, for compounds such as BZN, is the cause of severe side effects when used for treatment in patients [25]. The toxicity of **CX1** *in vitro* was over 500-fold greater than the IC_50_ suggesting that this compound may be developed into a therapeutic drug. However, as **CX1** is an amphiphilic compound, its cardiotoxicity will need to be evaluated carefully [26]. Additionally, this compound induced effective lysis of intracellular amastigotes, showing a strong trypanocidal activity. While trypanostatic drugs, such as **NT1**, may be more effective against the acute phase of disease, where parasites replicate rapidly, inducing lysis like **CX1** does might be essential for the development of drugs against the chronic stage of Chagas disease, where parasites are found in a quiescent intracellular state.

Finally, **PCH1** is characterized by a central hydrazide moiety that bridges a pyridine ring on the carbonyl side and furan and chlorophenyl rings on the nitrogen end. We observed that the position of the nitrogen in *ortho* within the ring is crucial for the effect, as well as the removal of the *meta*-chloride substituent. The hydrazide compound **PCH1** induced major changes in amastigote morphology, such as presence of larger amastigotes in which replication of DNA-containing organelles took place, but normal cytokinesis into daughter cells was abnormal. Several compounds that affect epimastigotes replication, such as the vinca alkaloids agents vincristine and vinblastine present a similar phenotype, with formation of giant cells containing multiple nuclei and kinetoplasts [27]. The microtubule stabilizing agent taxol also inhibits cell division, but, unlike treatment with **PCH1**, the parasites retain a normal nucleus/kinetoplast relationship [28]. At higher doses, **PCH1** however had a trypanolytic effect. Moreover, **PCH1** was found to have a deleterious effect on intracellular *L. major* with an estimated IC_50_ of ∼2 µM and was more potent against *L. amazonensis*, which replicates in a large communal phagolysosome, with an estimated IC_50_ of ∼100 nM. As hydrazide groups are problematic in a compound because of the possibility of release causing toxicity [29], attempts to replace this group with a bioisostere should be made during chemical optimization.

In conclusion, HTS assays are a good tool to identify new compounds with anti-kinetoplastid activity. In this study, we found three new compounds, all possessing hydrophobic groups including multiple aromatic rings, at least one of which being nitrogen-substituted. It is apparent that the most important feature of the three highly effective compounds is the presence of hydrophobic, aromatic moieties. However, it is further apparent that electronic effects also serve a critical role. Despite the chemical similarities observed, the different phenotypic changes induced by each compound suggest that they are affecting different pathways in the intracellular parasites. As we have demonstrated their efficacy *in vitro*, it is now critical to determine their toxicity in animals and their efficacy *in vivo* to assess their potential as therapeutic agents against Chagas disease and leishmaniasis.

## Supporting Information

Alternative Language Abstract S1 Translation of the Abstract into Spanish by Ana Rodriguez
(0.04 MB DOC)

Figure S1 (0.11 MB XLS)
