# Supplementary material for: Identification of Three Classes of Heteroaromatic Compounds with Activity against Intracellular Trypanosoma cruzi by Chemical Library Screening
Source: PLoS Negl Trop Dis. 2009 Feb 24;3(2):e384. doi: 10.1371/journal.pntd.0000384 (PMC2639639; doi:10.1371/journal.pntd.0000384)
Supplement: Alternative Language Abstract S1 — Translation of the Abstract into Spanish by Ana Rodriguez (0.04 MB DOC) [file pntd.0000384.s001.doc]

**Title and abstract translation to Spanish.**

**Título:** Identificación de tres clases de compuestos heteroaromáticos con actividad frente a *Tripanosoma cruzi* intracelular por muestreo de librerías químicas

**Resumen:** El desarrollo de nuevas drogas frente a la enfermedad de Chagas es una prioridad debido a que las medicinas disponibles en este momento tienen efectos tóxicos, eficacia parcial y están dirigidas frente a la fase aguda de la enfermedad. En este estudio, hemos optimizado un ensayo de células enteras para muestreo de alto rendimiento que inhibe la infección de células de mamífero por tripomastigotes de *T. cruzi*. Una librería química de 2000 compuestos fue analizada usando un *T. cruzi* (cepa Tulahuen) recombinante que expresa β-galactosidasa. Se seleccionaron tres compuestos por su alta actividad frente a *T. cruzi* y su baja toxicidad en células huésped *in vitro*: PCH1, NT1 y CX1 (IC_50_: 54, 190 y 23 nM, respectivamente). Cada uno de estos tres compuestos presenta un mecanismo de acción diferente sobre la proliferación intracelular de amastigotes de *T. cruzi*. CX1 muestra una alta actividad tripanocida, una característica esencial para el desarrollo de drogas frente a la fase crónica de la enfermedad de Chagas, donde los parásitos se encuentran en forma intracelular quiescente. NT1 tiene un efecto tripanostático, mientras que PCH1 afecta a la división celular. Los tres compuestos también muestran alta actividad frente a *T. cruzi* intracellular de la cepa Y y frente a las especies de kinetoplastidos relacionados *Leishmania major* y *L. amazonensis*. La caracterización de la actividad anti-*T. cruzi* de moléculas relacionadas químicamente con las tres seleccionadas inicialmente de la librería química ha permitido la selección de dos compuestos con valores de IC_50_ de 2 nM (PCH6 and CX2). Estos valores son aproximadamente cien veces mas bajos que los de las medicinas que se usan actualmente en pacientes frente a *T. cruzi*. Estos resultados proporcionan nuevas moléculas candidatas para el desarrollo de tratamientos frente a la enfermedad de Chagas y la Leismaniasis.

Translation of the Abstract into Spanish by Ana Rodriguez
